# Natural Products from Ethnodirected Studies: Revisiting the Ethnobiology of the Zombie Poison

**DOI:** 10.1155/2012/202508

**Published:** 2011-10-02

**Authors:** Ulysses Paulino Albuquerque, Joabe Gomes Melo, Maria Franco Medeiros, Irwin Rose Menezes, Geraldo Jorge Moura, Ana Carla Asfora El-Deir, Rômulo Romeu Alves, Patrícia Muniz de Medeiros, Thiago Antonio de Sousa Araújo, Marcelo Alves Ramos, Rafael Ricardo Silva, Alyson Luiz Almeida, Cecília de Fátima Castelo Almeida

**Affiliations:** ^1^Laboratory of Applied Ethnobotany, Department of Biology, Federal Rural University of Pernambuco, 52171-900 Recife, PE, Brazil; ^2^Laboratory of Pharmacology and Molecular Chemistry, Department of Biological Chemistry, Cariri Regional University, Pimenta 63105-000, Crato, CE, Brazil; ^3^Laboratory of Herpetology and Paleoherpetology, Department of Biology, Federal Rural University of Pernambuco, 52171-900 Recife, PE, Brazil; ^4^Laboratory of Ictiology, Department of Biology, Federal Rural University of Pernambuco, 52171-900 Recife, PE, Brazil; ^5^Ethnozoology, Conservation and Biodiversity Research Group, Department of Biology, State University of Paraíba, João Pessoa 58429-500, PB, Brazil

## Abstract

Wade Davis's study of Haitian “zombification” in the 1980s was a landmark in ethnobiological research. His research was an attempt to trace the origins of reports of “undead” Haitians, focusing on the preparation of the zombification poison. Starting with this influential ethnopharmacological research, this study examines advances in the pharmacology of natural products, focusing especially on those of animal-derived products. Ethnopharmacological, pharmacological, and chemical aspects are considered. We also update information on the animal species that reportedly constitute the zombie poison. Several components of the zombie powder are not unique to Haiti and are used as remedies in traditional medicine worldwide. This paper emphasizes the medicinal potential of products from zootherapy. These biological products are promising sources for the development of new drugs.

## 1. Introduction

Ethnopharmacological studies have been extensively discussed as a promising strategy for development and discovery of new medical and pharmaceutical products [1]. Ethnopharmacology relies on accumulated cultural experiences with nature to aid in identifying bioactive molecules. There is evidence that ethnographically directed studies may be more efficient than other bioprospecting strategies; however, most of these studies have focused on the use of plants [1]. Gertsch [2] has argued that many of the pharmacological activities attributed to natural products are merely artifacts from data extrapolations, particularly in *in vitro *studies [3], and unreliable assays. This suggests a basic question: why have pharmacological investigations of thousands of plants and animals yielded so few products? One of the main reasons is a disconnection between investment and research; the large investments made for screening these compounds are not matched by those for research.

These findings emphasize the diversity of molecules and commercial drugs that result from ethnographically directed investigations. Such research, despite its potential for developing new drugs, has not attracted significant amounts of investment. Coordinated efforts to advance this mode of drug discovery are in their initial stages. Significantly, increasing numbers of leads are derived from animal products [4], although ethnodirected efforts related to the fauna are still scarce.

Like plants, animals have been a source of medicinal treatments since antiquity. Their presence in the pharmacopoeia of traditional populations [5] is considered universal by many researchers. The hypothesis of universal zootherapy, for example, postulates that every human culture that has developed a medical system utilizes animals as a source of medicine [6].

The ubiquity of animals in folk medicine is illustrated by studies of ethnobiology worldwide [7–11]. These studies, which have attracted increasing academic interest in recent years, have found a great deal of diversity in the animals used for therapeutic purposes, including insects [12, 13], vertebrates [7, 14, 15], and marine invertebrates [16, 17]. Studies of medicinal animals can assist in pharmacological screening and may serve both as a source of medicine and as a measure of economic value for these species [18].

In a review of new drugs from natural products, Harvey [19] reported 24 drugs based on animals and 108 based on plants, which suggests that animals are still poorly studied. Moreover, many pharmacological studies of animals did not report use of ethnographic information [20, 21].

This work examines the state of ethnodirected pharmacology in two ways. First, based on anthropologist Wade Davis's 1986 description, the components of the traditional Haitian “zombie powder” poison were studied. For the animal species involved in the poison preparation, we examine nomenclatural changes, geographic distributions, origins, ecological data, and conservation status. For the plants involved, we briefly review their current ethnobotanical uses, phytochemistry, and pharmacology as measures of the progress made since the Davis' report. We also discuss how ethnographic information about animal-derived medicines is used in present-day pharmacology and the relative scarcity of animal-derived products in natural product libraries. This paper suggests that traditional knowledge about animals can assist in locating potential therapeutic agents and is expected to fill gaps in knowledge about the traditional use of this resource.

Published ethnopharmacological reports regarding medicinal animals and plants were analyzed. For animals, reports and secondary documents describing medicinal animal use and pharmacological reports describing the use of traditional zoological products were used. Documents were obtained from Science Direct (http://www.sciencedirect.com/), Scirus (http://www.scirus.com/), Google Scholar, Scopus (http://www.scopus.com/), Web of Science (http://www.isiknowledge.com/), and Biological Abstracts (http://www.science.thomsonreuters.com/) using the following search terms: “Zootherapy + Biochemistry,” “Ethnozoology + Bioactive compounds,” “Ethnozoology + Biochemistry,” “Medicinal animals,” “Ethnozoology + Pharmacology,” and “Ethnopharmacology + Animals.” Review papers were excluded. A database characterizing the general profile of the studies and aspects related to the animal's popular and pharmacological uses was assembled. In some cases, the studies dealt with pharmacological analyses of more than one animal species, but only provided information regarding the popular use of one. In these cases, only species with both pharmacological and popular use data were incorporated into the database.

Ethnobotanical, phytochemical, and pharmacological information describing each of the plant species described by Davis [22] was assembled. First, the Scirus database (http://www.scirus.com/), which includes Science Direct, MedLine, and PubMed and Google Scholar were searched using the search terms “[*scientific species name*] AND ethnobotanicals,” “[*scientific species name*] AND pharmacology,” “[*scientific species name*] AND chemical composition”, and “[*scientific species name*] AND molecules;” review articles were also considered. Patent records were searched using the Scirus database with the search terms “[*scientific species name*] AND medicine” and “[*scientific species name*] AND drugs.” For species with few related scientific studies, we also performed a supplementary search at the Natural Products Alert database (NAPRALERT). It is not our intention to present an exhaustive review of the secondary literature, but rather to give an overview of the literature pertaining to these species.

## 2. Ethnobiology of the Zombie Poison

### 2.1. Landmark: Wade Davis—The Serpent and the Rainbow

Travel narratives are a longstanding source of information about America's peoples and environment. Foreign visitors undertook the process of developing a nomenclature for the newly discovered regions. In the 1980s, writing in this mode, Davis contributed a landmark piece of ethnopharmacological research.

Davis was born in BC, Canada, in 1953. He studied at Harvard the University, where he graduated with a doctorate in ethnobotany. He studied various Indian tribes, providing him with wide-ranging experience and making him a renowned ethnobotanist and photographer [23]. He has written and continues to publish books and scholarly articles. One of his major contributions involves his ethnopharmacological study of “zombie poison.”

This research began after Davis had completed his studies at Harvard and returned there as an assistant to Richard Evans Schultes. Schultes, a professor at the Botanical Museum of Harvard, studied the ethnobotany of the Indians of Northwest Amazon. He was particularly interested in medicinal plants, particularly hallucinogens, seeing such study a possible source of new medicines.

In 1982, Schultes asked Davis to travel to Haiti to “initiate the search for the Haitian zombie poison” [22] and to develop research suggested by Nathan Kline, a psychiatrist studying psychopharmacology, and Heinz Lehman, the director of the Department of Psychiatry and Pharmacology at the McGill University. Kline told Davis, “if we could find a new drug that the patient became deeply insensitive to pain and paralyze him, and another to return him harmlessly to normal consciousness, this would revolutionize modern surgery” [22]. Lehman added, “That's why we meet to investigate all reports of potential anesthetic agents. We must look more closely at this supposed zombie poison, if it exists” [22]. Kline affirmed that the “undead” [22], or “zombies,” were victims of Vodou practitioners.

Davis hypothesized the existence of an anesthetic, which, administered in adequate dosage, would reduce the metabolism of the victim to the point that he or she would be considered dead. However, the victim would remain alive and could be revived with the administration of an antidote. Such a drug would have broad medical and pharmacological potential. At the time, this process even attracted the interest of NASA as a model of artificial hibernation [22].

Davis aimed to discover “the frontier of death” [22], as Lehman put it. He traveled to Haiti to find Voodoo practitioners and obtain samples of the zombie poison and antidote, observing Voodoo preparation and recording their use. Davis stood out among ethnobotanists for his work on the “zombification” process, in which he strove to be systematic and objective. His work described and contextualized the process, its mystique, and the animal and plant species involved. This research forms the foundations of our knowledge of the anesthetic contained in the zombie poison.

Davis' travel chronicle, *The Serpent and the Rainbow* [22], provided important insights and observations about this phenomenon. He described it as “an elusive phenomenon that [he] had difficulties to believe” [22]. His investigations of the zombie phenomenon were of great technical, scientific, and marketing relevance. Davis describes zombification in long passages of his narrative, leaving a rich commentary on what he saw.

### 2.2. Zombification: Theory and Practice

Since 1915, when Haiti was occupied by the United States of America, zombification has attracted interest in western culture [22, 24]. From the standpoint of western psychiatry, a “zombie” is defined as a female or male individual that has been poisoned, buried alive, and resurrected. These individuals manifest symptoms that would be classified as a catatonic schizophrenic, characterized by inconsistency and catalepsy, alternating between moments of stupor and activity [22]. As described by Davis, the word “zombie” had meaning rooted in the culture and beliefs of the Haitian peasant society. “*Precisely the Haitian definition of zombie [is of] a body without character, without will*” [22]; a zombie is “undead” [25] and in a state of lethargic coma. A zombie, in this sense of the word, is identified through its lifeless expression, nasal intonation, and repeated and limited actions and speech.

“Zombification” is a religious practice related to Voodoo. From the perspective of Voodoo, zombies are created by witchcraft, an essentially magical phenomenon. These beliefs regarding the natural and supernatural worlds developed over Haiti's history of colonization and intermarriage and are a synthesis of the religious beliefs of Haiti's original inhabitants with those of African origin and European Christianity [26, 27]. The zombie poison powder was controlled by Haitian secret societies with roots in West Africa. The poison was and still is used as a form of sanction for those who “violated the codes of society”. In Haiti, zombies are not themselves considered objects of fear; rather, popular fear focuses on becoming a victim of zombification [22, 28].

In Haiti, the estimated number of new zombifications exceeds one thousand cases per year [28]. Despite its apparent prevalence, Haiti classifies this practice as criminal activity tantamount to murder (Article 246 of the Criminal Code of Haiti).

In his publications, Davis suggested that zombies in Haiti were “produced, made,” in contrast to the image of folkloric zombies [29]. He showed the existence of zombies using the rational methods of western science, revolutionizing the ethnographic narrative when placed in the first person [29]. Reflecting on his research on the zombie poison, Davis said: “*[…] it is implied that its main chemical components had to be topically active. For descriptions of wandering zombies, it seemed likely that the drug induces a prolonged psychotic state, whereas the initial dose had to be capable of causing a stupor similar to death. Since, in all probability, the poison was an organic derivative, its source had to be a plant or an animal commonly found in Haiti. Finally, whatever was the substance, it should be of an extraordinary power*.”

Davis was especially interested in the plant and animal species used to prepare the poison: *“[carried] a kaleidoscopic Haitian bag built of empty cans of soda. The specimens that I filled included lizards, a polychaete worm, two marine fish and numerous tarantulas—all preserved in alcohol—as well as several bags of dried plant material. Two bottles of rum contained the antidote, while the poison itself was in a glass jar […]” *[22]. He later added: *“If the mystery of the zombie phenomenon had to be resolved, these specimens were the most important clues. Without them, there was nothing concrete.”*

Davis' descriptions of zombification continue to have great relevance as records of the process of bringing a person apparently dead to life and have come to play an important role in the pharmaceutical industry's understanding of ethnography as a source of new drugs. Within this context, Davis' publications provoked a great deal of controversy in the foreign press. Most reports suggested that his writing combined folklore, culture, ethnobotany, and pharmacology [29, 32]. Similarly, many reported, often with a tone of censure, that Davis caricatured Voodoo as a closed cultural system, ignoring changes since its formation in the eighteenth century [29, 33].

There are many descriptions of zombies and the practices surrounding zombification, ranging from scientific reports and doctoral dissertations to popular movies, computer games, magazines, websites, and numerous other forms of cultural expression [26, 34, 35]. References to zombies can even be found in computing, biotechnology, and artificial intelligence [29].

### 2.3. Composition of the Zombie Poison

As noted throughout the text, many interesting issues surround zombification. We have highlighted several of these issues in Davis' report. Davis' interdisciplinary approach included documenting the formulations of the zombie poison and antidote [24]. He helped to develop the field of ethnobiology by answering questions as an interdisciplinary ethnobiologist that could not be answered with other modes of inquiry.

During his field research in Haiti between 1982 and 1984, Davis learned of eight distinct zombie poison formulations and assisted in their preparation *in loco*. At the time, he had two main informants: Marcel Pierre, “*an old and faithful follower of Francois Duvalier*” [22], and Herard Simon.

From Pierre, Davis learned of a poison preparation that contained plant and animal material from five distinct species. The preparation related by Simon contained 15 species encompassed in 13 genera, and his account included the administration of a preparation based on *Datura stramonium* L. (Solanaceae) after zombification. Only Pierre revealed the composition of an antidote, which contained plant material from five species, including a plant only identified by its genus (see Table 1).

Here, we do not present a complete account of the scientific research surrounding zombification, nor do we address the controversy surrounding the “truth” of this practice. This paper instead aims to recover the composition of the zombie poison, as reported by Davis, and survey our knowledge of each of its components (with emphasis on animal-derived products) and its implications for the development of new drugs. Davis' narrative serves as a platform to discuss the utility of ethnobiological study.

## 3. Survey of the Current Components of the Zombie Poison and Antidote

### 3.1. Plants

Plant species are the most widely used sources in folk medicine, with thousands of species used around the world. (In some cases, the specific plant parts used in the poison have not been investigated in chemical and pharmacological studies. The specific plant parts used to prepare the zombie poison are not known for all species.)

#### 3.1.1. *Albizia lebbeck* (L.) Benth

Davis' informants cited *A. lebbeck* as a major component of the zombie poison. Pierre's preparation employed the fruit, while Simon's included the seeds [22]. In various populations in India, the juice of the roots of *A. lebbeck* combined with those of the leaves and bark of *Diospyros peregrine* is used to treat snake bites [36], asthma [37], diseases related to vision, night blindness, pyorrhea, toothache, insect and scorpion bites [38], disorders related to male fertility [39], wounds, leprosy injuries [40], and various inflammatory conditions [41].

Extracts of *A. lebbeck *stem bark contain tannins, flavonoids, anthraquinones, saponins, steroids, terpenoids, and coumarins [41, 42]. Ethanolic extracts and petroleum ether were tested against four models of inflammation in rats (carrageenan, dextran, Freund's adjuvant, and cotton pellet) and administered at a dose of 400 mg/kg. The substances gave inhibitions ranging from 34.46% to 68.57% [41]. The aqueous extract of *A. lebbeck* showed antimicrobial activity against nine different microorganisms [43]. The methanol extract of the bark administered to rats affected levels of testicular androgens by altering spermatogenesis [44], and the saponins present in the bark interfered with fertility in rats [45]. Antispermatogenic and antiandrogen activities are related [44, 46]. A protein called lebbeckalysin, isolated from the seeds, has antitumor, antibacterial, and antifungal activities [47]. There are dozens of patents involving this species and its chemical components (such as a set of herbs with antiallergenic properties, international publication number WO 2006067802 A1).

#### 3.1.2. *Aloe vera* (L.) Burm. f

The antidote described by Pierre included the leaves of the *Aloe vera *plant [22]. These leaves are used to treat leukorrhea [48], hypertension, heartburn, cancer, dandruff, stomach problems, hair loss, bruises, rheumatism, intestinal helminthes, and inflammation and are also used as an emollient [49]. This species is also used to aid in the healing process [50].

This plant contains diverse chemical compounds, including anthraquinones, carbohydrates, enzymes, proteins, vitamins, and hormones, of which several exhibit pharmacological activity [51, 52]. Extracts and chemical components of *A. vera* have shown immunostimulant, antimicrobial, antidiabetic, anti-inflammatory, wound healing, antioxidant, anticancer, hepatoprotective, and skin moisturizing effects, as well as utility in treating skin diseases [51, 52]. This species is used in pharmaceutical, hygienic, and cosmetic products [51]. Out of all the species discussed here, *A. vera *is represented in most patents. For instance, patent WO 2006055711(A1) describes a preparation containing *A. vera* for treatment of neurological syndromes, chronic pain, inflammatory bowel disease, and viral infections.

#### 3.1.3. *Anacardium occidentale* L

Simon cited *A. occidentale *as a component of the zombie poison [22]. This species is widely used in human food and is available commercially in processed food products. Davis [24] reported that this species was traditionally used as a purgative, diuretic, febrifuge, and cough treatment. The leaves and stem bark are used to treat diarrhea, kidney infection, heartburn, inflammation of the female organs, tuberculosis, general inflammation, and diabetes and was also used as an antiseptic [49].

*A. occidentale's* leaves contain tannins, flavonoids, and saponins [65]. The hydroalcoholic extract of the leaves did not produce toxic symptoms in rats at doses up to 2000 mg/kg [65] and showed antiulcer activity [66]. Anacardic acid, a phenolic compound, has not produced biochemical or hematological changes in rats at doses below 300 mg/kg [67] and has shown antioxidant activity [68] and cytotoxic activity against leukemic cells by inducing apoptosis [69]. Anacardic acid derivatives have been patented as antimicrobial agents (WO2008062436A2).

#### 3.1.4. *Mucuna pruriens* (L.) DC

The fruit of *M. pruriens* was employed in the poison described by Pierre [22]. In India, its seeds are ground with almonds and ingested to treat sexual debility and rheumatism and are also used as a tonic [38]. Haitians use these plants to treat parasitic infections; a teaspoon of the hair of the *M. pruriens* fruit (which contains formic acid and mucunaina) mixed with *Psidium guajava* L. is taken before breakfast for three days, causing severe diarrhea that eliminates worms from the intestine and stomach [70].

The seeds of *M. pruriens* contain alkaloids [71], phenolic compounds, tannins, L-dopa, lectins, protease inhibitors, and saponins [72]. This species has shown antioxidant and chelating activities [73]. The ethanolic extract of these seeds showed aphrodisiac activity in male rats with no adverse effects or ulceration at a dose of 200 mg/kg [74]. Clinical studies have shown that *M. pruriens* regulates steroidogenesis and improves semen quality in men with infertility [75]. Studies have shown this species to be an effective treatment for Parkinson's disease *in vivo*; this activity could be related to the presence of L-dopa, an important drug for the treatment of Parkinson's disease [76–78]. The aqueous extract of its seeds has shown hypoglycemic effects [79]. The seeds have also been shown to be beneficial treatments for venomous snake bites [80, 81]. *M. pruriens* extract has been patented for the treatment of Parkinson's (WO 2005092359 A1).

#### 3.1.5. *Prosopis julifiora* (Sw.) DC

*P. julifiora *was a component of the poison described by Simon [21]. Brazilian sources record the use of this species' leaves for the treatment of skin diseases [49], asthma, bronchitis, conjunctivitis [82] fever, warts, gonorrhea, eye problems, parasites, diarrhea, and ulcers [83–88].

This species contains steroids, alkaloids, coumarins, flavonoids, sesquiterpenes, and stearic acid [89]. The hydroalcoholic extract of its pollen had antioxidant activity both *in vivo* and *in vitro* [89]. The alkaloid fraction from its leaves has been observed to have significant effects on glial cells, inducing cytotoxicity, reactivity, and nitric oxide production [90]. Moreover, it has shown antipyretic, diuretic, antimalarial, antibacterial, hemolytic, and antifungal activities [85, 91–95]. No drug patents involving this species or its constituents were found.

#### 3.1.6. *Capparis cynophallophora* L

The leaves of *C. cynophallophora *were part of Pierre's antidote preparation [22]. It has been used to treat cough, pneumonia, flu, digestive problems, skin diseases, abdominal pain, rheumatism, snakebites, and digestive problems and has also been used as an emmenagogue [49, 96]. Two common flavonoids, kaempferol and quercetin, have been isolated from this species [97]. Oliveira et al. [9] reported that these flavonoids may exhibit antinociceptive activity [9]. This species was not found in any registered patents.

#### 3.1.7. *Zanthoxylum martinicense* (Lam.) DC

Simon's poison preparation included *Z. martinicense* [22]. Davis [24] reported that, in Cuba, the leaves and bark of this plant were used as a tonic and to treat syphilis, rheumatism, and alcoholism. It has also been reported to act as an antispasmodic, rheumatism treatment, diuretic, and narcotic [98, 99]. A phytochemical screening revealed the presence of isoquinoline alkaloids, triterpenes/steroids, lignans, quinones, lactones/coumarins, tannins/phenols, and saponins [100, 101]. The plant showed antifungal activity against two microorganisms, *Microsporum canis* and *Trichophyton mentagrophytes* [102]. There were no registered patents for this species.

#### 3.1.8. *Guaiacum officinale* L

*G. officinale* was used in the antidote described by Pierre [22]. Records of Caribbean natives using this species as a treatment for reproductive problems date back to the 16th century [103]. It has also been used to treat inflammation of the stomach, inflammatory diseases of respiratory organs, rheumatism, amenorrhea, and gonorrhea and has been used as a laxative, anticonvulsant, cardiac depressant, diuretic, diaphoretic, chronic, expectorant, abortifacient, diuretic, purifying treatment, and antidote for accidental poisoning [95, 104–110]. Its chemical composition includes triterpenes, alkaloids, and various guaianins [111, 112]. Its extracts have shown *in vitro* and/or *in vivo* stimulant activities for smooth muscle, as well as abortifacient, diuretic, antimicrobial, anti-inflammatory, and spasmolytic activities [113–117]. This species is included in several patents; in one example, its prepared extract was patented to treat skin inflammation and psoriasis (EP1832294A1).

#### 3.1.9. *Trichilia hirta* L

In Simon's account, *T. hirta* was used to prepare the poison [22]. Davis [24] reported the use of its leaves to treat anemia, asthma, bronchitis, and pneumonia and as a tonic in Cuba. It contains steroids and triterpenes [118–120]. Its methanolic extract showed no antibacterial activity against the organisms *Escherichia coli* and *Staphylococcus *[121], but antimalarial and larvicidal activities were reported [122]. This species was not found in any patents.

#### 3.1.10. *Petiveria alliacea* L

*P. alliacea* was cited by Simon as a component of the zombie poison [22]. A substance found in this species, dibenzyl trisulphide, exhibits antitumor and immunomodulatory activities [123]. The extract displayed several mechanisms of action that may explain its antitumor activity, such as cell cycle arrest in G2 phase, induction of cytoskeletal reorganization and DNA fragmentation [124]. The benzyl trisulfide and benzyldisulfide fractions of the plant's crude extract showed acaricidal activity in *Rhipicephalus* (*Boophilus*) *microplus* [125]. Several compounds isolated from this species have antibacterial and antifungal activities [126, 127]. The extract of *P. alliacea* showed promise as a wound treatment [128]. Fractions of the extract of this species also showed depressant activity in mice [129], and anti-inflammatory and analgesic effects have also been reported [130]. A product that includes the patented dibenzyl trisulphide compound was indicated for the treatment of cancer (20080070839 A1).

#### 3.1.11. *Urera baccifera* (L.) Gaudich

*U. baccifera *was cited by Simon as a component of the zombie poison [22]. It is used as an emmenagogue and to treat persistent fever, skin infections, snakebites, aches and pains, rheumatism, inflammation, arthritis, gastrointestinal disorders, and gonorrhea [85, 131–135]. It has anti-inflammatory and analgesic activities *in vivo* [136]. Its extract did not show pronounced leishmanicidal activity [135]. No patents relating to this species were found.

#### 3.1.12. *Cedrela odorata* L

*C. odorata *was a component of the zombie poison antiotde described by Pierre [22]. This astringent plant is used to treat pain, malaria, fever, aches, atonic seizures, anemia, gangrene, diarrhea, abdominal pain, chills, edema, vertigo, coughs, malaise, gastrointestinal pain, leishmaniasis, stroke, tooth pain, numbness after an insect bite, and erysipelas and is used as an abortifacient and vermifuge [63, 122, 137–141]. Several compounds have been isolated from this species, including sesquiterpenes, triterpenes, flavonoids, steroids, and limonoids [142–145]. No patents related to this species were found.

#### 3.1.13. *Dieffenbachia seguine* (Jacq.) Schott

*D. seguine* was among the components of Simon's zombie poison preparation [22]. This plant is considered toxic in many parts of the world. However, it is used as a choleretic, female aphrodisiac and contraceptive and to treat dropsy, gout, dysmenorrhea, sexual impotence, and sterility [98, 146–149].

Tannins, alkaloids, terpenoids, steroids [150], triterpenes, and a great variety of lipid compounds [151] have been reported in the extracts of *D. seguine's* leaves. These extracts showed weak antiproliferative activity on a human colon cancer cell line with IC_50_>50 *μ*g/mL [150]. The sap of this species contains toxic metalloproteins that cause necrosis at the site of contact. A patent describing the use of plant substances, including a substance from species *D. seguine*, as spermicidal and anti-infective agents and as prophylactics against sexually transmitted diseases and the human immunodeficiency virus has been filed (WO 2007074478 A1). Other studies have reported vasodilator, hypotensive, antifertility, contraceptive and/or interceptive, and spasmogenic activities [152–155].

#### 3.1.14. *Datura stramonium* L

In Simon's account, he indicated that *D. stramonium*, among other ingredients, was administered after removal of a zombie from the grave [22]. This species is commonly used to treat asthma and as a hallucinogen. Sixty-seven unique tropane alkaloids have been detected in its extract. At certain concentrations, this plant is known to induce delusions and altered mental states [156]. Agglutinin, a lectin isolated from *D. stramonium*, inhibited proliferation and induced differentiation in glioma cells [157]. There are thousands of patents related to scopolamine (a commercialized pharmaceutical product) directly or indirectly. One example of these is a European patent application for the treatment of depression and anxiety (WO 2006127418 A1).

#### 3.1.15. *Dalechampia scandens* L

*D. scandens* was a component of Simon's zombie poison preparation [22]. It is used to treat cough and flu [122], and cytotoxic activity has been reported [158]. No patents related to this species were found.

**Comments**

Plants have been the main source of molecules for the development of new drugs. Cragg et al. [159] reported that “more than 60% of anticancer agents used are derived from natural products.” Significant plant-derived medicinal substances include elliptinium, etoposide, irinotecan, taxol, vincristine, and teniposide, among others [159].

Generally, the components of the poison by Pierre and Simon [22] would be expected to have toxic effects, while those of the antidote might have beneficial effects (detoxifying, hepatoprotective, or immunomodulatory activities, e.g.).

*M. pruriens, P. alliacea, U. baccifera, D. seguine, *and* D. stramonium* were all cited as poison components. The seeds of *M. pruriens* have been shown to be effective in *in vivo* studies and clinical trials for the treatment of Parkinson's disease and contain a compound that is commercially exploited for this purpose [160]. The extract of *M. pruriens* and specifically L-dopa has proven effective in the treatment of many symptoms, such as tremor, difficulty in movement, difficulty walking, and depression, in the pathology of Parkinsons. *P. alliacea* and *U. baccifera* show analgesic activity [22]. *D. seguine* may facilitate the absorption of the bioactive substances of the poison, because this species causes irritation in the epidermis. The studies also indicate that *D. stramonium* is a potent hallucinogen; this activity may be due to anticholinergic activity triggered by its tropane alkaloids, such as hyoscyamine and scopolamine, which may be metabolized into atropine.

*A. vera* used as an antidote may be due to any number of its observed beneficial properties, including immunostimulant, antioxidant, and hepatoprotective activities.

A sizeable obstacle in understanding the pharmacological mechanisms and effects of the zombie poison is the disparate chemical and pharmacological components in its preparation. This diversity makes it difficult to disentangle each species' specific role. Such preparations combining traditional components often act on not only physiological but psychological and spiritual levels. Study of the role of each compound could provide clues about their roles in the poison.

Although the majority of plant species considered here have been the subject of at least one *in vitro* or *in vivo *pharmacological study, and they contain dozens of known bioactive molecules, research on the pharmacological basis of the process of zombification is not conclusive; the role of each of the molecules involved in this process is not yet known. Almost 30 years after Davis' research, there are still many unanswered questions.

### 3.2. Amphibians

Vertebrate species are among the least-used in folk medicine; approximately 29 species are known to be employed [161, 162].

#### 3.2.1. *Rhinella marina* (Linnaeus, 1758) (Buga Toad)

*Rhinella marina *(Linnaeus, 1758), also known as the common toad, large buga toad, or cane toad, has undergone several modifications in the genus and epithet (*Bufo marinus *Schneider, 1799;* Bufo marinus *Gravenhorst, 1829; *Bufo angustipes* Taylor & Smith, 1945; *Bufo pythecodactylus* Rivero, 1961; *Bufo marinus* Cei, Erspamer & Roseghini, 1968) but has always been classified within the family Bufonidae [163–166]. This species is native to Central America (Belize, Costa Rica, El Salvador, Guatemala, Honduras, Mexico, Nicaragua, Panama, and Trinidad and Tobago) and South America (Bolivia, Colombia, Ecuador, Guyana, French Guiana, Peru, Suriname, Venezuela, and the southern portion of Brazil) [166–170]. 

The buga toad was likely introduced into Haiti from explorers' ships or as a biological form of pest control [171, 172]. Because it is aggressive and highly dispersive, this toad is found in both natural and urban environments and is abundant everywhere it is found [173]. It is nocturnal in several ecosystems, including the Amazon rainforest, savanna, humid woodlands, equatorial dry forests, agroecosystems, and urban areas, with population peaks in open and altered areas [173]. It can be found in many microhabitats, such as leaf litter, holes in buildings, falling trees, branches, and leaves [174]. *R. marina's* rising population requires urgent conservation measures to prevent local extinction of native species, and it represents a major threat to frog fauna [164, 175, 176].

Species in the family Bufonidae derive toxic and pharmacological properties from granular glands in their backs [177], which biosynthesize several chemical compounds for protection from predators and microorganisms [178]. These properties make this family valuable as a source of bufotoxins, a class of bioactive molecules [179]. It is also a significant cause of injury for domestic and wild animals, mainly resulting from predation attacks [180]. Substances isolated from the skin of these toads, referred to as “dendrobatid alkaloids,” are used as antimicrobial agents, a chemical defense against predators, irritants, hallucinogens, convulsants, nerve poisons, and vasoconstrictors. The alkaloid epibatidine, a painkiller 200 times more potent than morphine, was also derived from this family, being found in some species of poison dart frogs. Other such alkaloids include batrachotoxins (sodium channel activators), histrionicotoxins (noncompetitive blockers of nicotinic channels), decahydroquinolines, various izidines, epibatidine (a potent nicotinic agonist), tricyclic coccinellines, pseudophrynamines, and spiropyrrolizidines (potent noncompetitive blockers of nicotinic channels) [181] and the pumiliotoxin, allopumiliotoxin, and homopumiliotoxin group.

#### 3.2.2. *Bufo bufo* (Linnaeus, 1758)

*Bufo bufo* (Linnaeus, 1758), popularly known as the common toad, is a complex of Bufonidae species. A review of the literature concerning these species is urgently needed, as *Bufo bufo* was synonymized with *Bufo vulgaris* (Laurenti, 1768), which became a null clade in conventional taxonomy [164, 166, 182, 183]. This species was observed first across almost all of Europe (except Ireland), most islands in the Mediterranean, the Middle East (Lebanon, Syria and Turkey), and North Africa (northern coast of Morocco, Algeria, and Tunisia) [182]. Although reported by Davis [22], it is not a native species of Haiti and was likely introduced during the colonization of the Hispaniola during the frequent contact with large vessels originating from the Iberian Peninsula, especially Spain. However, to the best of our knowledge, there are no taxonomic occurrences of this species in Haiti. *Bufo bufo* is a habitat generalist and is found in urban areas, coniferous forests, seasonal forests, woodlands, meadows, and arid environments [184]. It is remarkable for its stable populations in areas where it is endemic. It has therefore attracted little concern from the International Union for Conservation of Nature—IUCN, although it is classified as near-threatened in Spain due to a sharp decrease in its population from constant trampling and climate change [185–187].

#### 3.2.3. *Osteopilus dominicensis* (Tschudi, 1838)

*Osteopilus dominicensis *Tschudi, 1838, a member of the family Hylidae [164, 166, 188, 189], is endemic to Haiti and the Dominican Republic [188, 190, 191] and is found at altitudes from sea level to 2000 m [171, 172, 188, 192]. It is found in lentic water bodies in open environments, forests, and agroecosystems, especially on the edges of permanent or temporary ponds [189, 190]. Like all hylids, its arboreal habits are facilitated by its adhesive discs [177], and it commonly uses bushes as vocalization sites [189, 190]. It is remarkable for its stable populations in areas where it is endemic. It has therefore attracted little concern from the International Union for Conservation of Nature—IUCN, although some studies have detected reductions in some isolated populations [188, 189, 193] and have recommended protection of their reproductive sites as a primary conservation measure [189, 193, 194]. We found no reports of the chemical composition of its skin or pharmacological activity related to this species or species of phylogenetically related genera such as *Osteocephalus *and* Phyllodytes *[164].

**Comments**

For centuries, the skin of amphibians, especially those of the genus *Bufo*, has been used in traditional Chinese and Japanese medicine [129]. Gomes and Colleagues [129] reported that these skins provide a wide range of bioactive compounds with different therapeutic potentials, including antiprotozoal, antiviral, antineoplasic, cardiotonic, antiarrhythmic, antidiabetic, immunomodulatory, antibacterial, antifungal, sleep-inducing, analgesic, contraceptive, behavior-changing, wound healing, and endocrine activities (other than insulinotropic). The molecules identified include bufogenins, bufadienolides, or bufotoxins, which, interestingly, have chemical structures that interact with the cyanogenic glycosides present, for example, in the plant *Digitalis purpurea* [195]. These authors reported vasoconstrictor activity from the skin secretions of *Rhinella marina* in an experimental model of umbilical artery rings and placental vessels.

The quality and quantity of bufadienolides from this species, for example, vary significantly during ontogenetic development, especially in eggs [196]. Gao et al. [197] found at least 43 compounds in methanol extracts from the genus *Bufo*, including commercial samples. These authors reported, from various sources, that at least 100 compounds have been identified, including bufadienolides and indole alkaloids. Despite reports of potential oncological applications of these substances, their adverse effects, such as cardiotonic action [129], are a source of concern. This cardiotoxicity is widely known, having been recorded for many species, including *Bufo viridis* [198]. Although we have reservations about the records of *Bufo bufo* in Haiti, this species is widely used in traditional oriental medicine. Gao et al. [197] observed, through an analysis of geographical variations, that in many cases the chemical composition of *Bufo* venom did not meet the requirements of Chinese pharmacopoeia.

Davis' informants provided information about the amphibians used to prepare the zombie poison [22]. The two formulations are quite different, both in their components (Table 1) and modes of preparation. Davis reported that both poisons employed both the common toad and the marine toad. He was likely referring to *Bufo bufo* (because in his work, he refers to this species similarly in other contexts) and *Rhinella marina*. One of the poisons also included the skin of the frog *Osteopilus dominicensis*. The ingredients of this poison were highly diverse and caught Davis' attention [22]. From an ethnopharmacologic perspective, the desired activity may be obtained through such additions due to interactions among the drugs present. However, it is also possible that such additions were used only to give importance and status to the manufacturer of the poison, and do not impact its pharmacologic activity.

### 3.3. Fish

Fish are among the animals most frequently used in traditional folk medicine. At least 110 species of fish in Latin America are used in traditional medical systems [161]. 

#### 3.3.1. *Sphoeroides testudineus* (Linnaeus, 1758)

Known as the puffer fish, painted puffer fish, or pining puffer fish, *S. testudineus *is found in the western Atlantic from New Jersey to Santa Catarina and is the most abundant species on the Brazilian coast [199–201]. It lives in bays and estuaries, reaching and entering freshwater, and reaches 25 cm in total length [199]. It is reef-associated and may spend its entire life cycle in estuarine waters. It is very abundant in fish assemblages in estuaries and bays [202–204]. This species, like others from the family Tetraodontidae, can as, a defense mechanism, inflate its body through ingestion of water or air. It feeds mainly on crustaceans, mollusks, plants, and invertebrates [205]. It reproduces by external fertilization in open waters by placing eggs on substrates and has a mean total length of 13 cm at sexual maturity [206]. It contains tetrodotoxin, a potent ichthyotoxin found in its skin, liver, and gonads, where it acts as pheromone [207]. This potent neurotoxin, also known as “tetrodox,” blocks potential actions in nerves by blocking voltage-gated, fast sodium channels in nerve cell membranes, preventing affected nerve cells from firing. The biological actions of the tetrodox include paresthesias [208] of the lips and tongue, followed by sialorrhea, sweating, headache, weakness, lethargy, ataxia, tremors, paralysis, cyanosis, aphonia, dysphagia, seizures, dyspnea, bronchorrhea, bronchospasm, respiratory failure, coma, and hypotension. In affected organisms, cardiac arrhythmias may precede a complete respiratory failure and cardiovascular collapse [209].

#### 3.3.2. *Sphoeroides spengleri* (Bloch, 1785)

Like *S. testudineus, Sphoeroides spengleri *is also commonly known as the puffer fish or pining puffer fish. *S. spengleri *is distributed in the western Atlantic from Mass, USA to Sao Paulo, Brazil [199, 200]. It is found in shallow waters near the coast that are not exposed to freshwater and is common on reefs. This species has high levels of tetrodotoxin in its muscles, skin, and viscera that constitute a risk to its predators, while the levels in puffer fishes of the genus *Lagocephalus *are smaller, suggesting a lower risk. However, there are no concrete data on this type of poisoning [210, 211]. *S. spengleri *feeds on mollusks, crustaceans, and echinoderms and reaches 15 cm [199]. It is often consumed by fishermen along with species of *Lagocephalus laevigatus *[210, 212], although most are captured for fishkeeping. 

#### 3.3.3. *Diodon holocanthus* (Linnaeus, 1758)

Known as spiny puffer fish, *D. holocanthus *is a widely distributed species found in almost all tropical areas of the western Atlantic, from Florida to southern Brazil. This marine species is associated with living reefs and reaches 30 cm [199]. In Brazil, it has been recorded in both shallow and deep coral reefs and always within the substrate, although not in abundance [213]. Spiny puffer fishes are nocturnal and are benthopelagic adults and pelagic juveniles. This species lives alone and feeds on mollusks, sea urchins, and crabs [214]. Members of the family Diodontidae can inflate their bodies by ingesting water or air as a complementary defense mechanism to their spikes. *D. holocanthus* is used in fisheries and is of great importance in fishkeeping [215].

#### 3.3.4. *Diodon hystrix* (Linnaeus, 1758)

*D. hystrix*, like the species above, is also known as spiny puffer fish. It is found in tropical and temperate regions worldwide. In the western Atlantic, it can be found from Massachusetts to southern Brazil [199]. This fish reaches 60 cm, has a relatively long pelagic stage, and feeds at night, with a diet mainly consisting of clams, crabs, and sea urchins [199]. It inhabits marine environments and lives in coral reefs up to 50 m deep. It lives alone and has nocturnal habits. They feed on invertebrates like sea urchins, gastropods, and hermit crabs [214]. It is not normally used as food, is rarely fished, and is instead used mostly as a commercial fishkeeping species [215].

**Comments**

Saxitoxin (STX) and tetrodotoxin (TTX), obtained from the above species [216], are considered key components in inducing catalepsy or motor paralysis, fundamental actions of the zombie poison [28].

However, there is evidence that other neurotoxic and cytotoxic substances are involved in zombie poison [216]. Landsberg et al. [217], compiling information from prior reports, reported that while TTXs and STXs are chemically different, they produce similar biological responses in mammals, including tingling and numbness of the mouth, lips, tongue, face, and fingers, paralysis of the extremities, nausea, vomiting, ataxia, drowsiness, difficulty in speaking, and progressively decreasing ventilation efficiency. A comparison of these symptoms with the descriptions of zombification reinforces the hypothesis that these neurotoxins are responsible for the phenomenon.

In a recent review, Zimmer [31] described the mechanisms and actions of TTX on the cardiovascular system of mammals. These actions include, depending on the dose, bradycardia, hypotension, a rapid drop in blood pressure, cessation of breathing, and dissociation/cessation of ventricular contractions. There is a set of clinical criteria for diagnosing TTX exposure; Table 2 compares these symptoms to those symptoms reported by Davis [22] for cases of zombie poisoning. This comparison also reinforces the hypothesis that TTX poisoning plays an important role in zombification.

We do not intend to evaluate the claims on the zombification here, given the complexity and controversy surrounding the issue. As Littlewood and Douyon [28] suggested, there is no single explanation for zombies, but mental disorders and equivocal identification may be plausible explanations. These authors studied three cases of possible zombification occurring between 1996 and 1997 in Haiti. At least two of those cases were equivocal identifications in which families claimed to recognize a deceased relation. Genetic analysis in these two cases showed that the zombies had no kinship with the people who recognized them. On this topic, Littlewood and Douyon [28] wrote, “What is more difficult to understand is the apparent acquiescence of the “returned relative” not only to being a zombie but to being a “relative.”” Zombification is therefore a phenomenon that transcends psychopharmacologically and reminds us of the need for understanding social and cultural context. To varying degrees, traditional medicines worldwide interact with a web of relationships beyond medicine and physiology.

## 4. Medical and Pharmaceutical Implications

TTX has received more attention than other natural marine products due to its potent inhibition of sodium channels. Although biotoxins have been the subject of research for about 70 years, only in the past 10 has significant progress been made [218], as illustrated by the growing number of publications. Until 2007, formulations containing TTX had not been approved for use in the United States [218]. Several patents have been deposited, such as one owned by Wex Pharmaceutical Inc. for the use of TTX and STX in pain management.

Among amphibians, the most studied species is most likely *Rhinella marina* (Linnaeus, 1758), presumably due to its wide distribution and abundance. Until 2000, despite its continued use in traditional Chinese medicine, few studies had been conducted on its pharmacological properties, therapeutic potential, or toxicity [219]. However, interest in TTX and STX has considerably raised this species' profile. Bufadienolides have been reported as excellent cardiotonics and as a possible alternative to drugs available on the market [220]. Nevertheless, to the best of our knowledge, there is only one registered patent in the United States (936 063) for an antifungal and antimicrobial peptide derived from *Bufo bufo gargarizans* (now known as *Bufo gargarizans*) (Cantor, 1842).

The scenario sketched here shows the pharmacological potential of animal toxins. This potential invites scientific investment, especially in the cases above, which are widely distributed and abundant and also display promising and relevant pharmacological activities. Even considering the status that these animals gained from Davis' ethnobiological reports [22] and all the following controversy, progress is slow, despite the obvious potential for the development of new drugs. One issue is that many organisms are studied for their chemistry and biological activity from an ecological and evolutionary perspective. In the next section, we discuss how ethnographical knowledge of folk medicine can be employed, with specific reference to zombie poison.

## 5. Pharmacological Studies of Animals Used in Folk Medicine

Although research on animal use in folk medicine is still in its initial stages, it has intensified in recent years, especially in Latin America (mainly Brazil and Mexico), Africa, and Asia. These surveys have found an impressive number of animals used in folk medicine. At least 1,500 animal species are known to be used in traditional Chinese medicine [221], and at least 587 are used in Latin America [161]; these numbers are likely to increase dramatically with additional research.

Most medicinal animals used in traditional folk medicine are vertebrates, although significant quantities of invertebrates (mainly insects) are also used. In general, the groups with the largest numbers of medicinal species were mammals, birds, fishes, and reptiles. Amphibians comprise the least common group among medicinal vertebrates. Worldwide reviews report at least 165 species of reptiles [222], 101 species of primates [223], 55 species of bovidae [224], and 46 carnivorous mammals [14, 223] used in traditional folk medicine. 

Pharmacological approaches that test the activity of animal products based on traditional knowledge are relatively rare (Table 3). Few studies in the literature use this approach; most ethnopharmacological research is instead focused on plants [19]. However, it is possible that some studies have made use of ethnographic information but did not explicitly state this. 

These studies are largely related to animals used in developing countries, with Brazil and China being the targets of four investigations and the medicinal animals of India and Saudi Arabia the focuses of three and one study, respectively. Studies with this approach are more common in developing countries because such countries depend on natural products to satisfy their medical needs [225]. China and Brazil have been reported to have high rates of plant and animal use for medicinal purposes [225], as can be seen in our survey of natural products.

Interestingly, nine of the studies tested the therapeutic activities of products derived from vertebrates (Table 3), while only three works employed invertebrates. This discrepancy is surprising because the regulations regarding vertebrates, even for research purposes, are generally much more restrictive than those for invertebrates. Moreover, vertebrate conservation is a more pressing issue than that for invertebrates [226].

Animal-derived products were assayed most often for antimicrobial activity (4 studies) and inflammatory and antipyretic activities (3 studies each). These three indications are also among the most common tests of efficiency for plant-based therapeutic natural products [227–230]. These tests are common because they are relatively simple, easy to conduct, and inexpensive and because of the high frequency of natural products indicated to treat them; they are well-studied and, therefore, have led to a vast number of natural product treatments.

Despite the prevalence of antimicrobial activity in pharmacological studies, such activity was found only once in vertebrate studies; it was found commonly, however, in studies of invertebrates. The prevalence of antimicrobial activity in invertebrates may be due to syntheses of toxins and other substances that can damage bacterial membranes or decrease their ability to multiply [19]. Some herbivorous invertebrates (such as insects) can concentrate secondary plant compounds, which may contribute to their anti-microbial activity [19].

Moreover, plant substances with antipyretic and anti-inflammatory activities are used more commonly than those of vertebrate origin. In fact, studies have found compounds from plants, such as saponins and terpenoids, in the chemical composition of various animal venoms [231]. However, vertebrates can also concentrate those substances, although in many cases they are derived from insects or are present in their biological composition [15]. For example, tribes in South America apply poison from the skin of poison dart frogs (of the family Dendrobatidae) to arrow tips used for hunting. However, most of the alkaloids that are known to give these frogs their toxic properties are derived directly from arthropods in their diets, which are mostly made up of insects [232, 233].

It is noteworthy that, in studies of the venom of Anura, there has been little investigation of the correlation between diet and bioactive biosynthesis because most research related to frog diets is limited to identification of their prey [234–236]. Frog diets generally exceed their energy requirements, as they are also used to produce toxins. This behavior is evidenced by research conducted with the family Dendrobatidae clade, which is found predominantly in tropical region, that found significant production of toxins among anurofauna [177, 237].

Some of the most commonly used pharmacological assays include measurement of the rectal temperatures of mice (three studies), analysis of minimum inhibitory concentrations (MICs) (three studies), and tests of mouse paw and ear edemas (two studies each). These tests reflect the most studied therapeutic indications.

This survey emphasizes the great potential of medicinal products obtained from zootherapy. In a more critical analysis, although interesting biological activities have been found, in our view, none was sufficiently potent to suggest potential for drug development. Additionally, such research carries implications for conservation because the amount of biomass required for such studies is substantial, and some species are endangered or vulnerable. All these aspects should be taken into consideration when designing studies, ethnographically inspired or otherwise, of medicinal animals.

## 6. Final Considerations

In this paper, we emphasize the great potential for study of medicinal animals from traditional knowledge, given the large number of species used worldwide. There is strong evidence of significant and relevant biological activities in vertebrate and invertebrate animals. The ethnobiology of the zombie poison, which contains ingredients used in many folk medical traditions that have led to the development of medically and pharmacologically important drugs, is an objective illustration of the role of traditional knowledge in modern drug discovery.

Although we found few pharmacological studies directly derived from zootherapy or ethnozoological studies, such studies are promising sources of potential new drugs. Thus, we recommend additional effort to develop pharmacological discovery studies using traditional medicinal animals. However, to balance the scientific and social impacts of this research, knowledge of each species natural history is required. This information will allow sustainable use of each species, allowing natural recovery and recruitment. Species of the family Bufonidae, especially the genera *Bufo* and *Rhinella* (Amphibia), and fish of the families Tetraodontidae and Diodontidae are promising candidates for investigation, both for their toxicological potential and as models of biosynthesis. Their favorable properties include significant toxin production, efficient metabolic pathways, potent biological activity, and their relative abundance. Surprisingly, there are still few studies on these species, despite the two extensively studied toxins, TTX and STX. Dozens of substances, some with neurotoxic and cytotoxic activity, can be recovered from these species. The few patent applications found underline the need for change in research methodologies and the wealth of biochemistry that has yet to be discovered.

These natural products are used in traditional medical systems by peoples around the world. They are present in their practices and strongly embedded in their culture. Understanding these phenomena may also prove useful for understanding how these medical systems have developed, especially in situations with limited access to the resources of modern medicine.

## Figures and Tables

**Table 1:** Components of the poisons and antidotes used for zombification, as related to Davis by informants Marcel Pierre (MP) and Herard Simon (HS).

Local name	Species	Family
Poison—MP		
*Plants*		
Pois-Gratter/Mucuna	*Mucuna pruriens* (L.) DC.	Fabaceae
Tcha-tcha	*Albizia lebbeck *(L.) Benth.	Fabaceae
*Amphibian*		
Cane toad	*Rhinella marina* (Linnaeus, 1758)	Bufonidae
*Fishes*		
Crapaud de mer	*Sphoeroides testudineus *(Linnaeus, 1758)	Tetraodontidae
Fou-fou	*Diodon hystrix *(Linnaeus, 1758)	Diodontidae

Antidote—MP		
*Plants*		
Aloe	*Aloe vera* (L.) Burm. f.	Xanthorrhoeaceae
*Bois ca-ca*	*Capparis cynophallophora* L.	Capparaceae
*Bois chandelle*	*Amyris maritima* Jacq.	Rutaceae
*Cadavre gâte*	*Capparis* sp.	Capparaceae
*Cedar*	*Cedrela odorata *L.	Meliaceae
Roughbark Lignum-vitae	*Guaiacum officinale* L.	Zygophyllaceae

Poison—HS		
*Plants*		
Ave	*Petiveria alliacea *L.	Phytolaccaceae
Bayahonda	*Prosopis juliflora* (Sw.) DC.	Fabaceae
Bresillet	*Comocladia glabra *Spreng.	Anacardiaceae
Bwa piné	*Zanthoxylum martinicense* (Lam.) DC.	Rutaceae
Cana muda	*Dieffenbachia seguine* (Jacq.) Schott	Araceae
Consigne	*Trichilia hirta *L.	Meliaceae
Maman guêpes	*Urera baccifera* (L.) Gaudich. ex Wedd.	Urticaceae
Mashasha	*Dalechampia scandens* L.	Euphorbiaceae
Pomme cajou	*Anacardium occidentale* L.	Anacardiaceae
Tcha-tcha	*Albizia lebbeck *(L.) Benth.	Fabaceae
*Amphibian*		
Hispaniolan common treefrog	*Osteopilus dominicensis* (Tschudi, 1838)	Hylidae
Cane toad	*Rhinella marina* L.	Bufonidae
*Fishes*		
Fugu	*Sphoeroides testudineus *(Linnaeus, 1758)	Tetraodontidae
Fugu	*Sphoeroides spengleri *(Bloch, 1785)	Tetraodontidae
Fugu	*Diodon hystrix *(Linnaeus, 1758)	Diodontidae
Fugu	*Diodon holocanthus *(Linnaeus, 1758)	Diodontidae

“Postzombification paste”—HS		
*Plants*		
Zombie cucumber/stramonium	*Datura stramonium* L.	Solanaceae

**Table 2:** Clinical grading system for TTX poisoning as described by Fukuda and Tani [30] and modified by Zimmer [31].

Clinical grading system	Description of zombification [22]
*First,* “oral numbness and paraesthesia, sometimes accompanied by gastrointestinal symptoms (nausea)”	Digestive disorders with vomiting.

*Second,* “numbness of face and other areas, advanced paraesthesia, motor paralysis of extremities, incoordination, slurred speech, but still normal reflexes.”	—

*Third,* “gross muscular incoordination, aphonia, dysphagia, dyspnoea, cyanosis, drop in blood pressure, fixed/dilated pupils, precordial pain, but victims are still conscious.”	—

*Fourth,* “severe respiratory failure and hypoxia, severe hypotension, bradycardia, cardiac arrhythmia, heart continuing to pulsate for a short period.”	Pronounced breathing difficulties, pulmonary edema, hypertension, hypothermia, renal failure, and rapid weight loss.

**Table 3:** Survey of nine pharmacological studies based on folk knowledge of medicinal animals. *While the listed activity was detected, there is no indication that the compound is popularly used for this purpose.

Order	Family	Species	Popular name	Detected activities	Reference
Artiodactyla	Camelidae	*Camelus dromedarius*	Dromedary	Cytotoxicity	[53]
Carnivora	Ursidae	*Ursus thibetanus*	Bear	Anti-inflammatory, anticonvulsant, analgesic	[54]
Carnivora	Ursidae	*Ursus arctos*	Bear	Anti-hepatitis C	[55]
Galliformes	Phasianidae	*Pavo cristatus*	Peacock	Anti-snake venom	[56]
Haplotaxida	Megascolecidae and	*Lampito mauritii*	Earthworm	Anti-inflammatory, antipyretic	[57]
Isoptera	Termitidae	*Odontotermes formosanus*	Termite	Antimicrobial	[58]
Isoptera	Termitidae	*Nasutitermes corniger*	Termite	Antimicrobial	[59, 60]
Perissodactyla	Rhinocerotidae	*Diceros bicornis*	Rhino	Antipyretic	[61, 62]
Squamata	Teiidae	*Tupinambis merianae*	Tegu	Anti-inflammatory, antimicrobial	[63, 64]
